# Why are breastfeeding rates low in Lebanon? a qualitative study

**DOI:** 10.1186/1471-2431-11-75

**Published:** 2011-08-30

**Authors:** Mona Nabulsi

**Affiliations:** 1 Department of Pediatrics and Adolescent Medicine, Faculty of Medicine, American University of Beirut, Beirut-Lebanon

**Keywords:** Breast feeding, Qualitative research, Lebanon

## Abstract

**Background:**

Breastfeeding is a cost-effective public health intervention that reduces infant morbidity and mortality in developing countries. In Lebanon, breastfeeding exclusivity and continuation rates are disappointingly low. This qualitative study aims at identifying barriers and promoters of breastfeeding in the Lebanese context by exploring mothers' perceptions and experiences in breastfeeding over a one year period.

**Methods:**

We conducted focus group discussions in three hospitals in Beirut, Lebanon, and followed up 36 breastfeeding mothers with serial in-depth interviews for one year post-partum or until breastfeeding discontinuation.

**Results:**

Themes generated from baseline interviews revealed several positive and negative perceptions of breastfeeding. Longitudinal follow up identified insufficient milk, fear of weight gain or breast sagging, pain, sleep deprivation, exhaustion, or maternal employment, as reasons for early breastfeeding discontinuation. Women who continued breastfeeding for one year were more determined to succeed and overcome any barrier, relying mostly on family support and proper time management.

**Conclusions:**

Increasing awareness of future mothers about breast feeding difficulties, its benefits to children, mothers, and society at large may further promote breastfeeding, and improve exclusivity and continuation rates in Lebanon. A national strategy for early intervention during school years to increase young women's awareness may improve their self-confidence and determination to succeed in breastfeeding later. Moreover, prolonging maternity leave, having day-care facilities at work, creation of lactation peer support groups and hotlines, and training of doctors and nurses in proper lactation support may positively impact breastfeeding exclusivity and continuation rates. Further research is needed to assess the effectiveness of proposed interventions in the Lebanese context.

## Background

Breastfeeding is a cost-effective public health measure that significantly impacts infant morbidity and mortality in developing countries [1,2]. Breastfeeding is associated with reduced infant risks of infections, atopic dermatitis, asthma, obesity, diabetes types 1 and 2, childhood leukemia, sudden infant death syndrome, necrotizing enterocolitis; and with higher Intelligence Quotient and academic performance at 6.5 years of age [3-5]. Moreover, it is associated with decreased maternal risks of diabetes type 2, breast and ovarian cancers, and postpartum depression [3].

In Lebanon, infant mortality rate remains high at 27/1000 births, with high morbidity. Death in the first month accounts for half of under-five mortality, and is partly blamed on the poor nutritional and health status of mothers and infants [6]. In addition, breastfeeding rates are disappointingly low with initiation rates varying between 63.8% and 96% [7,8]. Exclusive breastfeeding is reported in 58.3% of babies less than one month, and in 10.1% to 4.1% of 6-month old infants [9-12]. Only 27.1% of one-year old infants continue to breastfeed [9]. Moreover, no national programs exist to promote breastfeeding practices or support lactating women, and maternity leave is limited to 40 days post-partum.

Predictors of low breastfeeding rates in Lebanon include lower socio-economic status, Caesarean birth, urban residence, early hospital discharge, mother's religion, male paediatrician, and hospital practices that hinder breastfeeding like lack of rooming in of mother and baby, fixed newborn feeding schedules, and offering glucose and water or artificial formula as first feeds instead of breast milk [8,10,13]. All previous studies however were cross-sectional and failed to explore maternal perceptions or experiences of breastfeeding.

In view of the positive impact of breastfeeding on maternal and infant health [3-5], the low breastfeeding rates reported from Lebanon, and the limitations of available studies, we conducted this qualitative longitudinal study to explore maternal perceptions and experiences of breastfeeding mothers using a series of interviews over one year, hoping to identify barriers and promoters of breastfeeding in the Lebanese context.

## Methods

### Design

We adopted a qualitative research approach to explore mothers' perceptions, feelings and experiences of breastfeeding, by conducting focus group discussions and in-depth interviews.

### Settings

Maternity wards in three hospitals in Greater Beirut were chosen because they served communities with different socio-economic, cultural, and religious backgrounds: the American University of Beirut Medical Center (AUBMC), Hotel Dieu De France Hospital (HDF), and Sahel General Hospital (SGH).

### Participants

We used theoretical sampling to recruit our participants from the participating hospitals. Women delivering healthy live term newborns were approached on their first postpartum day by research assistants who visited the hospitals on daily basis during the recruitment period, and invited mothers to participate in the study after explaining its aims and procedures. We believe that recruiting participants from this setting will help us understand the perceptions and experiences of breastfeeding mothers in our context.

### Data collection

The study was done in two stages. In the first stage, we conducted one focus group discussion in each hospital using an interview schedule composed of five open-ended questions (Appendix 1), to test and improve the questions for use in the second stage. Discussions took place between November and December 2007, with 3-4 participants per group for a total of 10 mothers. A research assistant qualified in qualitative research methods conducted all discussions in colloquial Arabic, with probing as needed. Discussions took 60 to 90 minutes, were tape-recorded and later transcribed.

In the second stage, we performed a series of in-depth interviews with 36 mothers recruited between January and May 2008 (12 participants from AUBMC, 10 from HDF, and 14 from SGH). Participants were followed up for one year or until breastfeeding was stopped, whichever occurred earlier, to explore breastfeeding barriers and promoters in a woman's natural environment that may affect her breastfeeding decisions. Baseline interviews were conducted prior to hospital discharge, lasted between 30-60 minutes each, were tape-recorded, and later transcribed. Follow-up interviews were brief, ranging from 10 to 30 minutes, conducted monthly for the first six months, and then every other month for the remaining six months. They were either telephone, or face-to-face interviews at participants' homes. During each contact, we asked about breastfeeding status and the reasons for continuing or stopping. Telephone calls were considered as in-depth interviews, despite their limitations in capturing participants' body language or contextual factors at home that may affect breastfeeding experiences. During both study stages, we returned to the participants for validation of findings.

### Data analysis

Transcripts of focus group discussions and interviews were analyzed using inductive thematic analysis [14]. Recurrent themes emerging from raw data were identified and summarized on spread sheets. Subsequently, major and minor themes were generated to provide insight into women's perceptions and experiences of breastfeeding. The interview process was stopped when saturation of themes was reached. We also calculated proportions of breastfeeding mothers at different time points during the study. For breastfeeding definitions, we adopted the WHO definitions of exclusive breastfeeding, breastfeeding initiation and breastfeeding continuation [15].

### Ethical Considerations

The study was approved by the Institutional Review Board at AUBMC, and hospital administrators of HDF and SGH. A written informed consent for interviewing and tape-recording was obtained from each participant. Participants were assured confidentiality and anonymity of recordings, transcripts and behaviors observed any time during the study, the voluntariness of participation and withdrawal, and that gathered information would be used solely for the purpose of the study.

## Results

During the study, 7 mothers withdrew or were lost to follow up, 3 stopped breastfeeding soon after hospital discharge, 8 prior to six months, and only 11 (35%) continued breastfeeding for one year. Breastfeeding continuation rates were highest among mothers recruited from AUBMC (61.5% at 6 months; 54% at 1 year) followed by SGH (33% at 6 months; 27% at 1 year). Interestingly, mothers recruited from HDF had the lowest continuation rates (20% at 6 months; 0% at 1 year) despite being the only Baby Friendly hospital. Similarly, six-month breastfeeding exclusivity was highest among AUBMC mothers (61.5%), followed by SGH (20%) and lowest by HDF mothers (0%).

The major themes generated from focus group discussions and in-depth interviews are described below:

### Breastfeeding is a joyful and connected experience

Mothers (34/46) described breastfeeding as a *joyful, entertaining, relaxing *experience that strengthens mother-baby emotional bonding and attachment:

..a beautiful feeling!...relaxing...your private time with baby away from the world.. as if you're walking in nature...your relation with him becomes stronger... he is happier, comfortable and secure. I loved it so much and would wait for the time to breastfeed! (Mother of two)

Breastfeeding provided great tenderness enabling mothers to tolerate any associated discomfort:

Initially, it is painful...after a difficult pregnancy I thought I will not care much for this baby. Yet, when I put her on my breast, I felt the tenderness. Breastfeeding brings baby and mother closer, especially when she latches on the breast...despite your pain, you tolerate it for the sake of your baby..(Mother of two)

... a mother not breastfeeding cannot be a mother because she cannot feel her baby and her baby cannot feel her..they will be strangers..when he's on your breast, he feels your tenderness and love, will know that you're his mother.. Any woman can bottle-feed but only with breastfeeding he will feel the affection! (First-time mother)

### Naturalness and benefit

Mothers acknowledged that breastfeeding is the best nutrition for babies because of its naturalness:

Breast milk is natural whereas formula is artificial..as if you're cheating on yourself! You don't know how is artificial milk made and of what chemicals! (First-time mother)

Most participants were aware of breastfeeding benefits to mother and child:

*Breastfeeding is beneficial for a mother's health..her uterus returns to normal size quickly..she also loses weight..*.

It decreases the risks of breast, uterine and ovarian cancers..how can I not breastfeed?

*.. my son who breastfed longer is stronger and more intelligent than his brother who breastfed for 3 months..also my daughter, breastfed for 2 years is stronger and more intelligent..I don't know if it's breast milk!*.

### Breastfeeding changes figure and breast shape

Despite their knowledge of its advantages, some mothers expressed fears pertaining to the negative effects of breastfeeding on their breasts and figure: (14 mothers):

..breastfeeding may change the breasts' shape and result in sagging....(Mother stopping at 1 month)

..about ¾ of women who don't breastfeed are scared for their figure and beauty, although it's not true! (Mother of three; breastfed all children)

I breastfed my baby exclusively..but became fatter..gained so much weight and became depressed.. You are instructed to eat well when breastfeeding.. I ate and became much fatter..then decided to stop breastfeeding and go on diet..The husband may not care about your weight problem but may start looking at other women..(Mother of two)

### Having bad or harmful milk

There were beliefs that maternal illness, stress, drug intake, or even pregnancy would cause *bad *or *harmful *milk:

I had a cold... the doctor prescribed cold medicine and I was scared it might harm the baby... (Mother of two)

..a frightened or stressed mother's milk is bad.. I stopped breastfeeding at 4 months..I was very frightened then [war] and they told me I should not breastfeed..bad luck.. (Mother of two who breastfed her first baby for 2 years)

### Breastfeeding is painful and tiring

Negative breastfeeding experiences seemed to have a significant impact on a mother's decision not to continue breastfeeding. Ten participants described their experience as painful, tiring and difficult; requiring frequent, day and night, time-consuming feeds, and resulting in maternal sleep deprivation:

You are told about all the positives of breastfeeding! No one tells you about the severe pain, or the struggle to make her latch on your breast...as you are unable to breastfeed, the baby cries more and more, and you become frustrated!.. it is very distressing! (First-time mother)

It is difficult and tiring..feeding every 2 hours... you're worried and lost..don't know if he is satiated, or if positioning is correct...(Mother of two)

Breastfeeding is tiring, waking up at night! I don't know how mothers do it but it is really very exhausting.. from 10:30 pm till 12:30 am and she barely took a little!...it takes a lot from the mother...all the time it has to be me, no one can replace me except when she's asleep... Yes, sleep deprivation is a barrier! (First-time mother)

I'm tired, not eating well, and don't have time .. my milk decreased..I have to work at home but prefer to take care of her..it is unreasonable to leave her and go eat..My mother in law helps with house work but no one helps with the baby! (First-time mother)

Mastitis, or sore nipples, resulting in painful breastfeeding may force a mother to stop breastfeeding:

I had sore nipples and my breast bled with pieces of nipple coming off. It started soon after breastfeeding initiation and continued thereafter.. I insisted on breastfeeding so that my baby will have good immunity..pain was so severe I cried loudly! My doctor prescribed different creams...finally I became very sad, depressed..it was God's will.. I had twins previously who breastfed for 1 year..this baby!.I am very sad to stop despite my will..(Mother of three; stopped at one month due to lack of appropriate professional advice)

### Mother's milk is insufficient

Insufficient milk was reported by 15 mothers who had to supplement their babies with artificial milk:

I am breastfeeding and supplementing with formula because I feel he is still hungry..he continues to cry so I give the bottle to make him sleep.. bottle is easier...a mother's milk may be insufficient or non-nutritious..(Mother of three)

One mother described how routine hospital practices interfered with breastfeeding her hospitalized baby:

They put him in intensive care for 12 days and was bottle-fed...then they allowed me to breastfeed but my milk decreased and he refused my breast...he got used to the bottle!

### Stopping breastfeeding because of work

Maternal employment constituted an important barrier for many mothers; especially because maternity leave in Lebanon is only 40 days. The job barrier is best described by this second-time mother and teacher who stopped breastfeeding her first child at two months to return to work. This time, determined to breastfeed for a longer time, she took an additional two-month leave to pump and store breast milk for later. Despite having a supportive husband and family, she became very tired, could not cope and finally stopped at three months:

*It is very difficult..I'm like a robot! I have a 4-year old who goes to school, plus my work at home, and my job... I stopped breastfeeding because of work..*.

### Maternal will and unselfishness

Despite the negative experiences, some women continued breastfeeding for one year. The theme recurring most in interviews with those participants was their preparedness for breastfeeding, and determination to face any difficulty. They showed deep conviction of the importance of breastfeeding and its benefits to mother and baby. Whether they faced pain, sleep deprivation, tiredness, house work or job, they overcame obstacles through proper time management, family support and determination. They often identified maternal *will *and *unselfishness *as being key factors for successful breastfeeding:

A mother should not think of herself, her comfort or sleep. She needs to think of the baby before herself. I moved my job to a location next to where my baby is so that I can teach for 2 hours and then breastfeed. He never took the bottle. I also minimized my working schedule..(A working mother of 3 children)

Initially, she didn't know how to take the breast. For 1 month, I kept trying without sleeping... I pumped even if I had to throw milk away, so that my breasts continue giving milk. After a month, her mouth grew bigger and she could breastfeed directly..at the end, there has to be a way! (A mother of two children; left her job to be with her children)

A mother needs to be aware that she has a new life now, that the baby will need time and will change her daily routine.. she should be ready to make that sacrifice.. some don't accept the change and feel that breastfeeding is restrictive, so they don't breastfeed. (A mother who breastfed all four children, pumped breast milk for her hospitalized newborn)

*Being a working mother should not be a barrier..one can store breast milk or support with additional formula, but not stop breastfeeding completely...*.

### Perceptions of family and society

Half of the participants were concerned that breastfeeding may cause excessive weight gain or breast sagging. Whereas some were explicit about this personal concern, others stated that it was the perception of society, friends or family:

Many of my friends do not breastfeed.. they're concerned it makes their breasts bigger or causes weight gain..my sister, like my friends, believes it too...they believe there is no difference between breast milk and artificial milk (First-time mother)

The belief that breast milk was insufficient to achieve satiety in the baby, and that a bottle is necessary to ensure satiety and a good night sleep seemed to be transmitted from one generation to another:

My mother did not have milk and none of my siblings were breastfed. My mother-in-law too, none of her four children were breastfed..and my sister also did not have milk to breastfeed her 2 boys.. it is familial.. some families are like that... (Mother of two)

Family perceptions of breastfeeding can influence a mother's decision to breastfeed:

..my husband liked the idea of breastfeeding, but grandparents prefer the bottle because baby remains hungry with breast milk..they think I don't have enough milk because my breasts are small..that my milk is dilute and not nutritious..(Mother of two)

### Family support

On the other hand, family support of a breastfeeding mother is expected in our culture and acts as an important promoter:

Our eastern society encourages breastfeeding because it is nutritious and results in bonding with baby..when family members see you breastfeeding they become happy, and you will be happy too: your mother, sister, husband ..they're all happy because you're breastfeeding (Mother of four)

When I go back to work, I will not be able to breastfeed..you cannot concentrate on the baby 100%..you need to take care of your work, your home or find someone you can leave the baby with. Thanks God I have my mother! (A recently divorced mother of three)

## Discussion

We found low breastfeeding rates among our participants consistent with findings of previous studies from Lebanon [7-12]. Barriers to breastfeeding included negative breastfeeding experiences, or negative perceptions of breastfeeding, whether at the personal, family or society levels. Misconceptions like insufficiency of breast milk to achieve satiety in the baby, and breastfeeding causing maternal weight gain or breast sagging were common reasons for early discontinuation, similar to what has been reported in studies from other countries [16-18]. Despite their knowledge of breastfeeding benefits, mothers stopping early were psychologically unprepared for breastfeeding-associated pain, sleep deprivation, exhaustion, or other dramatic life changes. Those difficulties were compounded by maternal employment, inadequate family support, or lack of professional advice, which are known barriers to breastfeeding success [19-22]. In contrast, mothers continuing for one year showed solid determination and preparedness to face all difficulties [19]. Their strong belief in breastfeeding, good time management skills, and family support empowered them to succeed in breastfeeding.

Our study is limited by the fact that participants were sampled from an urban setting. Hence, women from rural areas may have different perceptions and experiences that need to be explored in future research. Also, telephone interviews are inferior to face-to-face interviews in capturing body language, and family interactions. Despite its limitations, our study sheds light on important, previously unexplored barriers to breastfeeding in the Lebanese context that should be addressed by public health professionals and policy makers.

## Conclusions

In designing national strategies to promote exclusive prolonged breastfeeding, it may be helpful to target young women early during school years to increase their awareness of the difficulties encountered by a breastfeeding mother, and competency in overcoming problems. Preparing future mothers for successful breastfeeding later in life may improve their determination and self confidence. In addition, developing national policies and programs that support breastfeeding mothers such as prolonging maternity leave, having day-care facilities at work, creating lactation peer support groups and hotlines, and training of doctors and nurses in proper lactation support, especially in relation to nipple care and maternal drug intake may improve continuation and exclusivity rates. Further research is needed to assess the effectiveness of proposed interventions in the Lebanese context.

## Competing interests

The authors declare that they have no competing interests.

## Authors' contributions

The author is responsible for the design, grant writing, literature review, data analysis, interpretation and manuscript preparation.

## Appendix 1

### Interview Schedule

a. What can you tell me about yourself?

b. What are your feelings about breastfeeding?

c. Can you tell me about your experience with breastfeeding?

d. What are some issues that, in your opinion, helped you go through the process of breastfeeding?

e. Can you tell me about issues that, in your opinion, hampered the breastfeeding process?

f. What are the opinions of people around you regarding breastfeeding like your husband, mother, mother-in-law, sisters, and friends?

g. Do you know whether you were breastfed?

h. How did you find your stay in the hospital?

i. Do you have anything more to add?

## Pre-publication history

The pre-publication history for this paper can be accessed here:

http://www.biomedcentral.com/1471-2431/11/75/prepub

## References

[B1] Molbak K Gottschau A Aaby P Hojlyng N Ingholt L Da Silva APJ Prolonged breastfeeding, diarrhoeal disease, and survival of children in Guinea-Bissau BMJ 1994 308 1403 6 8019249 10.1136/bmj.308.6941.1403 PMC2540341

[B2] WHO Collaborative Study Team on the Role of Breastfeeding on the Prevention of Infant Mortality Effect of breastfeeding on infant and child mortality due to infectious diseases in less developed countries: a pooled analysis Lancet 2001 355 451 455 10841125

[B3] Ip S Chung M Raman G Chew P Magula N DeVine D Trikalinos T Lau J Breastfeeding and maternal and infant health outcomes in developed countries Evid Rep Technol Assess 2007 153 1 186 PMC4781366 17764214

[B4] Kramer MS Chalmers B Hodnett ED Sevkovskaya Z Dzikovich I Shapiro S Collet JP Vanilovich I Mezen I Ducruet T Shishko G Zubovich V Mknuik D Gluchanina E Dombrovskiy V Ustinovitch A Kot T Bogdanovitch N Ovchinikova L Helsing E PROBIT Study Group (Promotion of Breastfeeding Intervention Trial) Promotion of breastfeeding intervention trial (PROBIT): a randomized trial in the Republic of Belarus JAMA 2001 285 413 20 10.1001/jama.285.4.413 11242425

[B5] Kramer MS "Breast is best": the evidence Early Hum Dev 2010 86 729 732 10.1016/j.earlhumdev.2010.08.005 20846797

[B6] World Health Organization (WHO) and UNICEF Revised Estimates of Maternal Mortality: A New Approach by WHO and UNICEF 1996 Geneva, Switzerland. World Health Organization

[B7] Ministry of Public Health Lebanese Republic National Perinatal Survey 1999 10

[B8] Batal M Boulghourian C Breastfeeding initiation and duration in Lebanon: are the hospitals "Mother Friendly"? J Pediatr Nurs 2005 20 53 59 10.1016/j.pedn.2004.09.004 15834361

[B9] Ministry of Social Affairs Pap Child Survey 1996

[B10] Batal M Boulghourian C Abdallah A Afifi R Breast-feeding and feeding practices in a developing country: a national survey in Lebanon Public Health Nutr 2006 9 313 319 10.1079/PHN2006860 16684382

[B11] World Health Organization (WHO): Global data bank on infant and young child feeding http://www.who.int/nutrition/databases/infantfeeding/countries/lbn.pdf

[B12] Chakar Rabay H Sokhn M Azar M Breast-feeding practice in a localized area of Beirut LMJ 1997 45 84 89 9289505

[B13] Al-Sahab B Tamim H Mumtaz G Khawaja M Khojali M Afifi R Nassif Y Yunis KA For the National Collaborative Perinatal Neonatal Network (NCPNN) Predictors of breastfeeding in a developing country: results of a prospective cohort study Public Health Nutr 2008 11 1350 1356 10.1017/S1368980008003005 18702836

[B14] Ritchie J Spencer L Micheal Huberman, Matthew B Miles Qualitative data analysis for applied policy research The Qualitative Researcher's Companion 2002 Sage Publications: Thousand Oaks 305 32921884488

[B15] World Health Organization (WHO) Indicators for assessing infant and young child feeding practices. Part 1 Definitions http://whqlibdoc.who.int/publications/2008/9789241596664_eng.pdf

[B16] Foster SF Slade P Wilson K Body image, maternal-fetal attachment and breastfeeding J Psychosom Res 1996 41 181 184 10.1016/0022-3999(96)00035-9 8887831

[B17] Barnes J Stein A Smith T Pollock JI Extreme attitudes to body shape, social and psychological factors and a reluctance to breast-feed. ALSPAC Study Team. Avon Longitudinal Study of Pregnancy and Childhood J R Soc Med 1997 90 551 559 9488013 10.1177/014107689709001007 PMC1296597

[B18] Arora S McJunkin C Wehrer J Kuhn P Major factors influencing breastfeeding rates: mother's perception of father's attitude and milk supply Pediatrics 2000 106 e67 10.1542/peds.106.5.e67 11061804

[B19] Manhire KM Hagan AE Floyd SA A descriptive account of New Zealand mothers' responses to open-ended questions on their breast feeding experiences Midwifery 2007 23 372 381 10.1016/j.midw.2006.01.002 17126458

[B20] Kools EJ Thijs C Kester ADM de Vries H The motivational determinants of breast-feeding: predictors for the continuation of breast-feeding Prev Med 2006 43 394 401 10.1016/j.ypmed.2006.03.012 16872670

[B21] Barona-Vilar C Escribá-Agϋir V Ferrero-Gandía R A qualitative approach to social support and breast-feeding decisions Midwifery 2009 25 187 194 10.1016/j.midw.2007.01.013 17493716

[B22] Huang PH Chen CH Wang HH The comparison of breastfeeding attitude and social support among pregnant women choosing different feeding methods J Nurs Res 2000 8 395

